# Effect of curcumin on inflammatory markers and disease activity in patients with rheumatoid arthritis: A meta-analysis

**DOI:** 10.1097/MD.0000000000046177

**Published:** 2025-11-28

**Authors:** Fuhan Zhang, Ben Niu

**Affiliations:** a Department of Rheumatology and Immunology, Guangdong Provincial Hospital of Traditional Chinese Medicine, Hainan Hospital, Haikou, Hainan, China; b Emergency Surgery Department, Guangdong Provincial Hospital of Traditional Chinese Medicine, Hainan Hospital, Haikou, Hainan, China.

**Keywords:** C-reactive protein, curcumin, Disease Activity Score in 28 joints, erythrocyte sedimentation rate, meta-analysis, randomized controlled trial, rheumatoid arthritis

## Abstract

**Background:**

Curcumin has been proposed as a potential adjunctive anti-inflammatory therapeutic option in rheumatoid arthritis (RA); however, the evidence regarding its clinical efficacy remains inconsistent. This meta-analysis aimed to evaluate whether curcumin supplementation improves disease activity and systemic inflammatory parameters in patients with RA.

**Methods:**

Following Preferred Reporting Items for Systematic Reviews and Meta-Analyses criteria, PubMed, Cochrane Library, Web of Science, and Embase were searched from inception to August 26, 2025. Eligible randomized controlled trials included adult RA patients diagnosed according to American College of Rheumatology or European League Against Rheumatism criteria and compared curcumin in any formulation with placebo or standard care. Two independent reviewers conducted study selection, data extraction, and methodological quality assessment using the Cochrane Risk of Bias 2.0 tool. Pooled analyses were conducted using Review Manager, version 5.4 and Stata, version 18, with fixed-effect or random-effects models based on heterogeneity.

**Results:**

Seven randomized controlled trials were included. Curcumin significantly reduced Disease Activity Score in 28 joints (weighted mean difference [WMD] −1.47; 95% confidence interval [CI] −1.68 to −1.26), rheumatoid factor (WMD −24.15; 95% CI −36.47 to −11.83), erythrocyte sedimentation rate (WMD −31.26; 95% CI −58.59 to −3.93), and C-reactive protein (WMD −0.93; 95% CI −1.33 to −0.53). Substantial heterogeneity was observed; nevertheless, leave-one-out sensitivity analyses confirmed stability of pooled estimates, and Egger tests revealed no significant publication bias.

**Conclusion:**

Curcumin supplementation was associated with statistically and clinically relevant improvements in disease activity and systemic inflammatory markers in RA. These findings support curcumin as a promising adjunctive therapeutic strategy, though larger, exposure-verified trials with extended follow-up remain required to validate long-term treatment effects and inform clinical implementation.

## 1. Introduction

Rheumatoid arthritis (RA) is a chronic, systemic autoimmune disease characterized by persistent synovial inflammation, progressive cartilage and bone destruction, and ultimately, functional disability. It affects ~0.5% to 1% of the global population, with a higher incidence in women and an onset typically between the ages of 30 and 50. The disease is associated not only with joint damage but also with significant extra-articular manifestations, including cardiovascular, pulmonary, and metabolic complications, which contribute to increased morbidity and premature mortality. Despite advances in pharmacological treatment, RA continues to impose a substantial burden on patients’ quality of life and healthcare systems worldwide.^[1,2]^ The introduction of conventional synthetic disease-modifying antirheumatic drugs (csDMARDs), such as methotrexate, and the subsequent development of biologic DMARDs and targeted synthetic DMARDs have significantly improved disease outcomes. However, not all patients achieve sustained remission, and many experience adverse drug reactions, high treatment costs, or diminished therapeutic responses over time. These limitations underscore the need for complementary therapeutic strategies that are safe, accessible, and effective in modulating inflammation and disease activity.^[3–5]^

Curcumin, a bioactive polyphenolic compound derived from the rhizome of *Curcuma longa* (turmeric), has attracted increasing attention as a potential adjunct in RA management. Extensive preclinical evidence demonstrates that curcumin exerts immunomodulatory and anti-inflammatory effects through multiple molecular mechanisms. It suppresses activation of the nuclear factor-kappa B pathway, downregulates pro-inflammatory cytokines such as tumor necrosis factor-alpha (TNF-α), interleukin-1 beta, and interleukin-6 (IL-6), and modulates cyclooxygenase-2 and inducible nitric oxide synthase expression. Furthermore, curcumin influences the Janus kinase/signal transducer and activator of transcription signaling cascade and enhances the expression of anti-inflammatory mediators, contributing to a broad immunoregulatory profile.^[6,7]^ Nevertheless, current clinical evidence is inconsistent. Variations in study design, sample size, intervention duration, curcumin formulation, and concomitant background therapies contribute to heterogeneity in results. A critical challenge lies in curcumin’s low oral bioavailability due to poor solubility, rapid metabolism, and limited systemic distribution. To overcome this, novel formulations, including nanoparticle-encapsulated curcumin, liposomal preparations, and co-administration with piperine, have been developed, though their relative efficacy in RA populations remains incompletely characterized.^[8–10]^

Given the expanding but heterogeneous body of literature, a comprehensive synthesis is warranted to clarify the effect of curcumin on inflammatory markers and disease activity in RA. Meta-analysis allows for quantitative integration of available evidence, improving statistical power to detect clinically relevant differences and exploring sources of variability across studies. Such evidence may inform clinicians, patients, and policymakers about the role of curcumin as an adjunctive anti-inflammatory agent in RA management and guide the design of future high-quality trials.

## 2. Methods

### 2.1. Search strategy

The present systematic review and meta-analysis was conducted in accordance with the Preferred Reporting Items for Systematic Reviews and Meta-Analyses guidelines.^[11]^ A comprehensive search was performed in PubMed, The Cochrane Library, Web of Science, and Embase from their inception to August 26, 2025, without language restrictions; non-English publications with available English abstracts were also considered. To ensure the completeness of the evidence base, reference lists of relevant studies, reviews, and clinical trial registries were manually screened to identify additional eligible trials. The search strategy combined Medical Subject Headings (MeSH) terms and free-text keywords, including “Curcumin,” “Turmeric,” “Curcuma longa,” “Rheumatoid Arthritis,” “RA,” “Arthritis, Rheumatoid,” “Randomized Controlled Trial,” “Clinical Trial,” and “Controlled Study,” which were integrated using Boolean operators and adapted to the indexing systems of each database. Detailed search strategies for each database are provided in Table S1, Supplemental Digital Content, https://links.lww.com/MD/Q776.

### 2.2. Inclusion criteria and exclusion criteria

Studies were included if they met the following criteria: participants were adults diagnosed with rheumatoid arthritis (RA) according to recognized diagnostic criteria (e.g., the American College of Rheumatology [ACR] or the European League Against Rheumatism [EULAR] classification criteria); the intervention group received curcumin, turmeric, or *Curcuma longa* extracts in any formulation or dosage, either alone or in combination with standard therapies; the control group received placebo, standard pharmacological therapy, or no intervention; outcomes included at least one measure of inflammatory markers (e.g., C-reactive protein [CRP], erythrocyte sedimentation rate [ESR], TNF-α, and IL-6) or disease activity indices (e.g., Disease Activity Score in 28 joints [DAS28]). Studies enrolling specific RA subgroups (e.g., patients with comorbid chronic periodontitis) were included only if they met standard ACR/EULAR diagnostic criteria for RA and reported relevant inflammatory or disease activity outcomes. These studies were retained to preserve comprehensiveness but were carefully examined for potential bias in sensitivity analyses.

Studies were excluded if they met any of the following criteria: they were reviews, case reports, editorials, conference abstracts, or animal/experimental studies; they lacked a control group or did not involve curcumin as an intervention; they did not report sufficient data for extraction and synthesis of effect sizes; or they included participants with comorbid autoimmune or inflammatory diseases other than RA, which could confound outcomes.

### 2.3. Literature screening and data extraction

Data extraction was carried out independently by 2 investigators who screened studies according to the predefined inclusion and exclusion criteria. Titles and abstracts were initially reviewed to exclude clearly irrelevant studies, and the full texts of the remaining articles were retrieved for further evaluation. Any discrepancies in study selection or data extraction were resolved through discussion; if consensus could not be reached, a third investigator was consulted to provide adjudication. For each eligible study, relevant data were extracted using a standardized form. Extracted information included the first author’s name, year of publication, country of origin, characteristics of study participants (including sex distribution, mean or median age), sample size, intervention details (formulation, dosage, and duration of curcumin therapy), comparator characteristics (placebo, standard treatment, or no intervention), and treatment strategies. In addition, key clinical outcomes were collected.

### 2.4. Quality assessment

The risk of bias of the included studies was assessed independently by 2 investigators. Each reviewer evaluated the methodological quality of the eligible studies, and the results were cross-checked for consistency. In cases of disagreement, consensus was reached through discussion, and if necessary, a third reviewer was consulted to resolve the discrepancy. For randomized controlled trials, the Cochrane Risk of Bias Tool, version 2.0 (RoB 2.0) was applied to assess the methodological rigor. This tool evaluates potential sources of bias across the following domains: bias arising from the randomization process, bias due to deviations from intended interventions, bias due to missing outcome data, bias in the measurement of the outcome, and bias in the selection of the reported result. Each domain was judged as “low risk of bias,” “some concerns,” or “high risk of bias,” and an overall risk-of-bias judgment was subsequently determined for each study.^[12]^

### 2.5. Statistical analyses

Meta-analyses were performed using Review Manager (RevMan) version 5.4 and Stata version 18 software. Statistical heterogeneity among studies was assessed by the chi-square test and quantified using the *I*² statistic. When *P* > .1 and *I*² ≤ 50%, a fixed-effect model was applied, indicating homogeneity across studies. Conversely, when *P* < .1 and *I*² > 50%, substantial heterogeneity was considered present, and a random-effects model was used for data synthesis. Continuous variables were analyzed using the standardized mean difference, while dichotomous variables were analyzed using the odds ratio. All effect sizes were reported with 95% confidence intervals (CIs). Publication bias was assessed using Egger linear regression test. The methodological quality of the included studies was evaluated with Review Manager version 5.4.1.

## 3. Results

### 3.1. Search results and study selection

**Figure 1.**
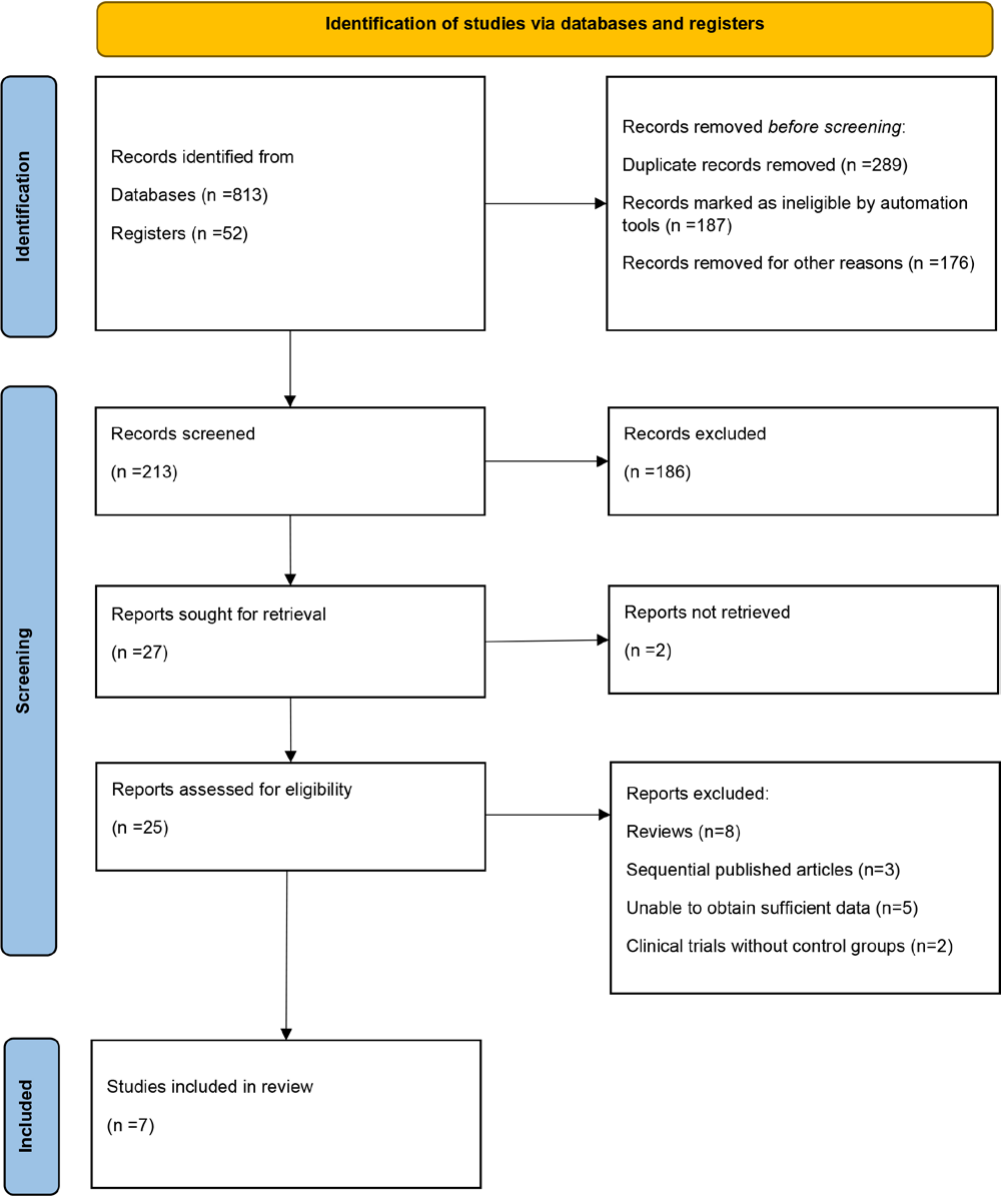
PRISMA flow diagram of the study selection process. PRISMA = Preferred Reporting Items for Systematic Reviews and Meta-Analyses.

A total of 865 records were initially identified through database (n = 813) and register (n = 52) searches. After removing duplicate records (n = 289), excluding records marked as ineligible by automated tools (n = 187), and eliminating those removed for other reasons (n = 176), 213 records remained for screening. Following the title and abstract screening process, 186 records were excluded, leaving 27 reports for retrieval. Of these, 2 reports could not be retrieved, and the remaining 25 were assessed for eligibility through full-text review. After detailed evaluation, 18 reports were excluded: 8 were reviews, 3 were sequentially published articles, 5 lacked sufficient data for analysis, and 2 were clinical trials without control groups. Ultimately, 7 studies met the inclusion criteria and were incorporated into the final meta-analysis^[13–19]^ (Fig. 1).

### 3.2. Study characteristics

A total of 7 randomized controlled trials were included in this meta-analysis, conducted between 2012 and 2022 across India and Iran. The sample sizes of the included studies ranged from 24 to 90 participants. The mean age of patients varied between 38.2 and 55.0 years, with some studies reporting age ranges (e.g., 45–60 years). The proportion of female participants differed across studies, from 41.7% to 100%, with several trials exclusively enrolling female patients. All included studies investigated the effects of curcumin in patients with rheumatoid arthritis, although the formulations, dosages, and treatment durations varied. Interventions ranged from conventional curcumin capsules (250–500 mg daily), nanomicelle preparations (40 mg 3 times daily), and high-dose regimens (180 mg/day administered 3 times per week), to combination therapies with standard anti-inflammatory drugs such as diclofenac sodium. Comparators included placebo, standard treatment, or combination therapy. The duration of interventions spanned from 8 weeks to 12 weeks, with 1 study extending to 90 days (Table 1).

**Table 1:** Characteristics of studies included in the meta-analysis.

First author	Year	Country	Mean age (yr) ± SD/range	Sample size (n)	Female, n (%)	Treatment strategies
Zarandi^[15]^	2022	Iran	50.5 ± 9.7	44	44 (100%)	Patients were treated with curcumin (500 mg once daily) or placebo for 8 wk.
Khan^[17]^	2022	India	45–60 yr	90	NR	Ninety patients were randomized into 2 groups. Curcumin was administered at 180 mg/day orally, distributed across 3 sessions per week.
Jacob^[19]^	2019	India	45–60 yr	24	12 (50%)	Patients were randomized in a 1:1:1 ratio to receive 250-mg curcumin, 500-mg curcumin, or placebo once daily for 3 mo.
Javadi^[16]^	2019	Iran	55.0 ± 2.9	49	44 (89.8%)	Patients received curcumin nanomicelle (40 mg) or placebo capsules 3 times daily for 12 wk.
Anusha^[18]^	2019	Iran	42.9 ± 4.8	45	45 (100%)	A triple-blinded controlled trial among 45 female patients with rheumatoid arthritis randomized into 3 treatment groups with different curcumin regimens.
Amalraj^[13]^	2017	India	38.2 ± 8.5	36	15 (41.7%)	Twelve patients in each group received placebo, 250 mg, or 500 mg of curcumin twice daily for 90 d.
Chandran^[14]^	2012	India	47.9 ± 12.0	45	38 (84.4%)	Patients were randomized into 3 groups to receive curcumin (500 mg), diclofenac sodium (50 mg), or a combination regimen.

NR = not reported, RA = rheumatoid arthritis, SD = standard deviation.

### 3.3. Quality assessment results

**Figure 2.**
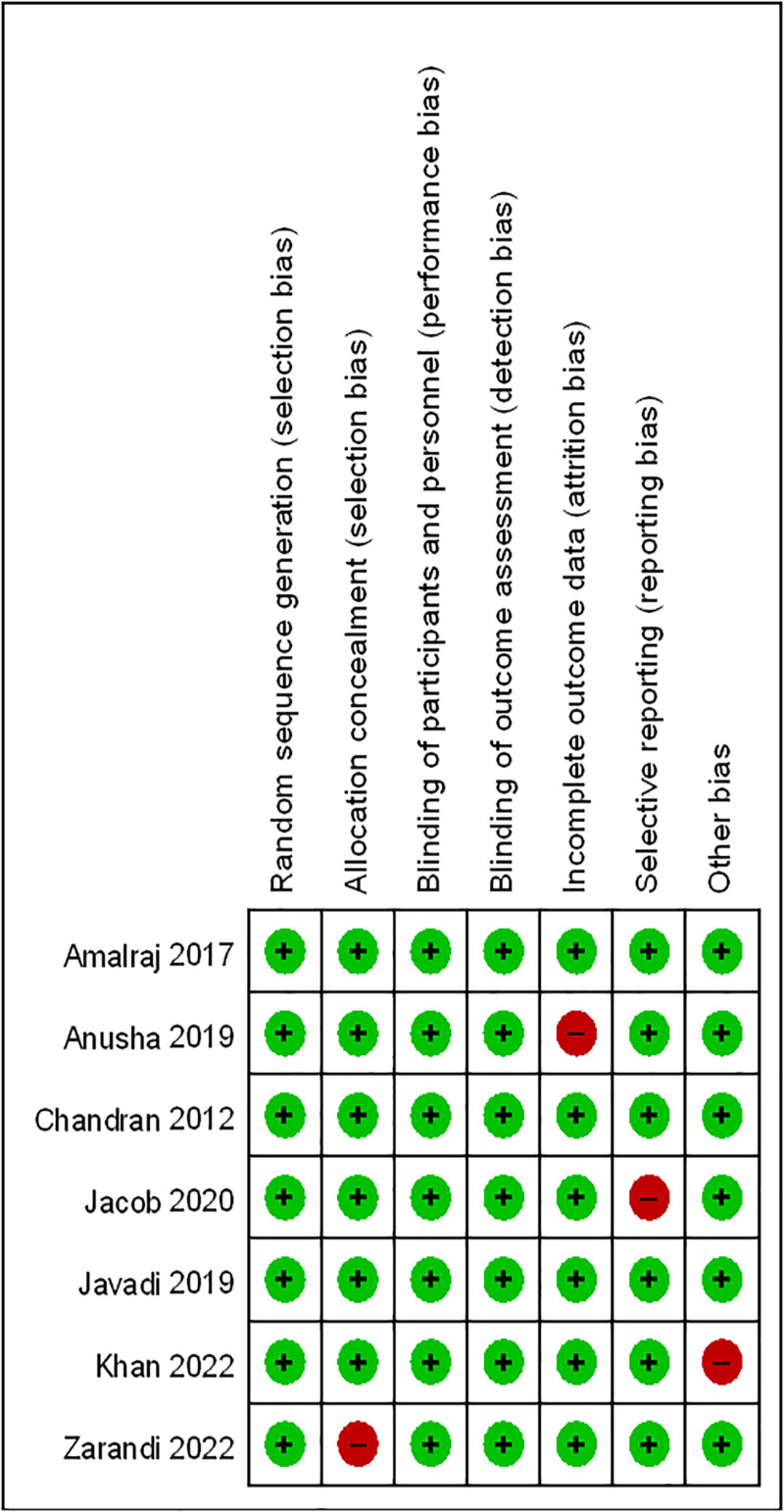
Risk-of-bias assessment of the included randomized controlled trials using the RoB 2.0 tool.

The methodological quality of the included randomized controlled trials was generally acceptable. Most studies demonstrated a low risk of bias across key domains, including random sequence generation, allocation concealment, blinding of participants and personnel, blinding of outcome assessment, and selective reporting. A few studies exhibited concerns in specific areas, particularly incomplete outcome data and allocation concealment, indicating potential risks of attrition bias and selection bias. Despite these limitations, the overall risk-of-bias profile suggested that the majority of the included trials were of moderate to high methodological quality, supporting the reliability of the synthesized evidence in this meta-analysis (Fig. 2).

### 3.4. Disease Activity Score in 28 Joints

**Figure 3.**
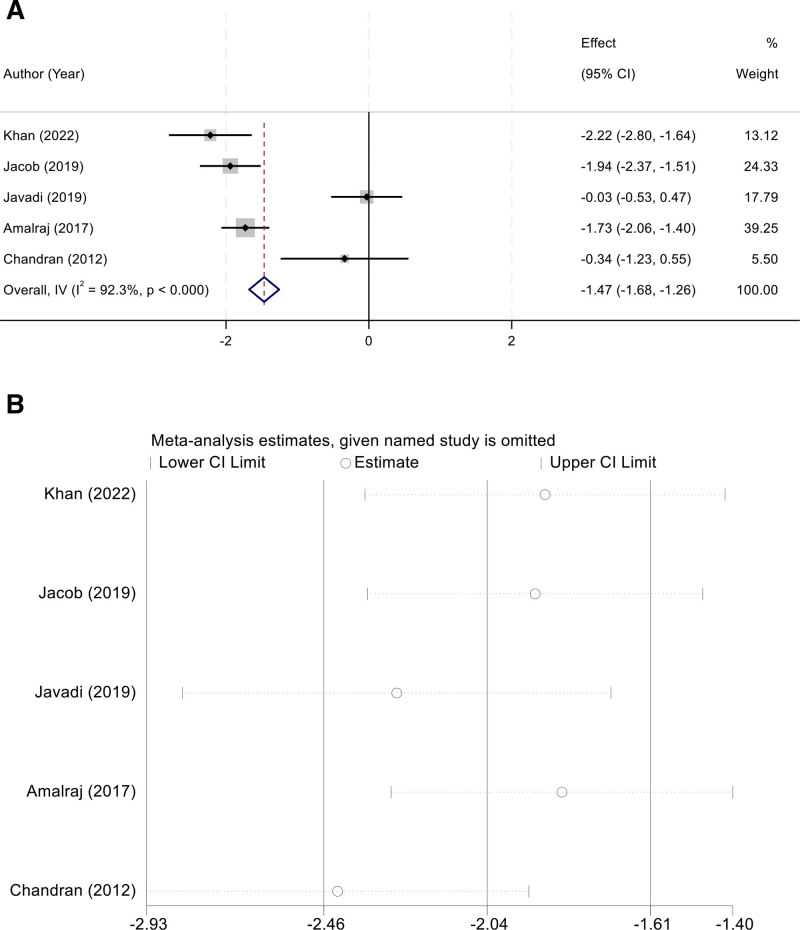
(A) Random-effects forest plot of WMD for DAS28 comparing curcumin versus control (negative favors curcumin); (B) leave-one-out sensitivity analysis demonstrating a stable pooled effect. WMD = weighted mean difference.

Five studies reported the effect of curcumin on DAS28 scores in patients with rheumatoid arthritis. Substantial heterogeneity was observed across studies (I² = 92.3%, *P* < .001), and therefore a random-effects model was applied. The pooled analysis demonstrated that curcumin supplementation significantly reduced DAS28 compared with controls (WMD = –1.47, 95% CI [–1.68,–1.26]) (Fig. 3A). Sensitivity analysis, conducted by sequentially excluding each study, confirmed that the overall results remained stable, suggesting that no single study disproportionately influenced the pooled estimate (Fig. 3B).

### 3.5. Rheumatoid factor

**Figure 4.**
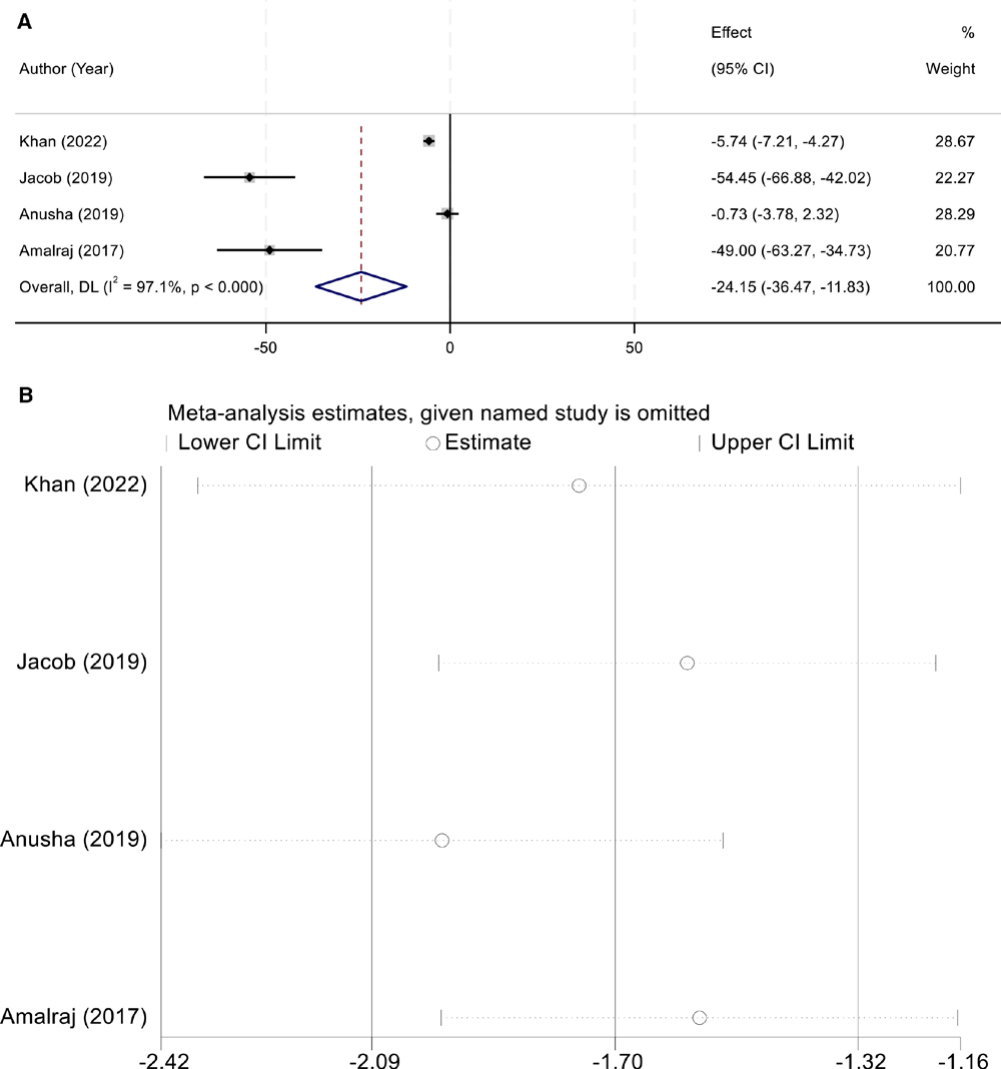
(A) Random-effects forest plot of WMD for RF (negative favors curcumin); (B) leave-one-out sensitivity analysis confirming robustness of the pooled estimate. RF = rheumatoid factor, WMD = weighted mean difference.

Four studies assessed the effect of curcumin on rheumatoid factor (RF) levels. Marked heterogeneity was detected among these studies (I² = 97.1%, *P* < .001), leading to the use of a random-effects model. The meta-analysis revealed a significant reduction in RF levels in patients receiving curcumin compared with controls (WMD = –24.15, 95% CI [–36.47,–11.83]) (Fig. 4A). Sensitivity analysis indicated that the pooled results were robust, with no evidence of substantial fluctuation after omitting individual trials (Fig. 4B).

### 3.6. Erythrocyte sedimentation rate

**Figure 5.**
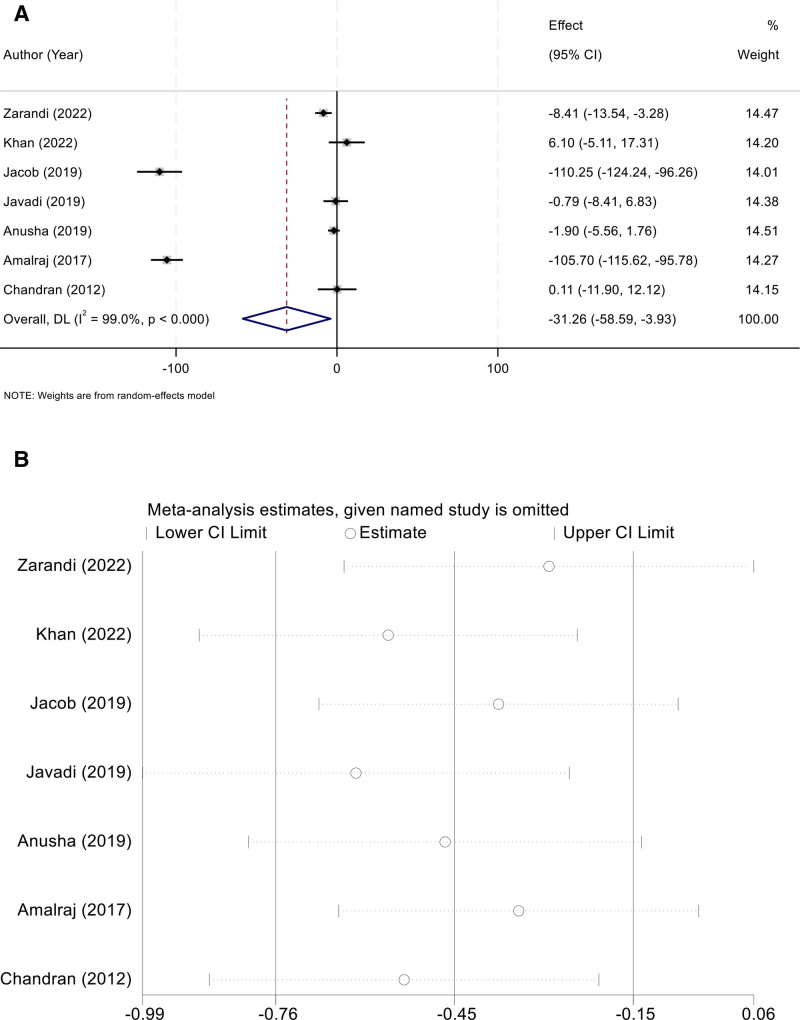
(A) Random-effects forest plot of WMD for ESR (negative favors curcumin); (B) leave-one-out sensitivity analysis indicating consistent results across iterations. ESR = erythrocyte sedimentation rate. WMD = weighted mean difference.

Seven studies reported ESR outcomes. A high degree of heterogeneity was identified (I² = 99.0%, *P* < .001), necessitating the use of a random-effects model. The combined analysis demonstrated that curcumin significantly decreased ESR levels compared with controls (WMD = –31.26, 95% CI [–58.59,–3.93]) (Fig. 5A). Sensitivity analysis showed consistent results after stepwise exclusion of individual studies, indicating the robustness of the findings (Fig. 5B).

### 3.7. C-reactive protein

**Figure 6.**
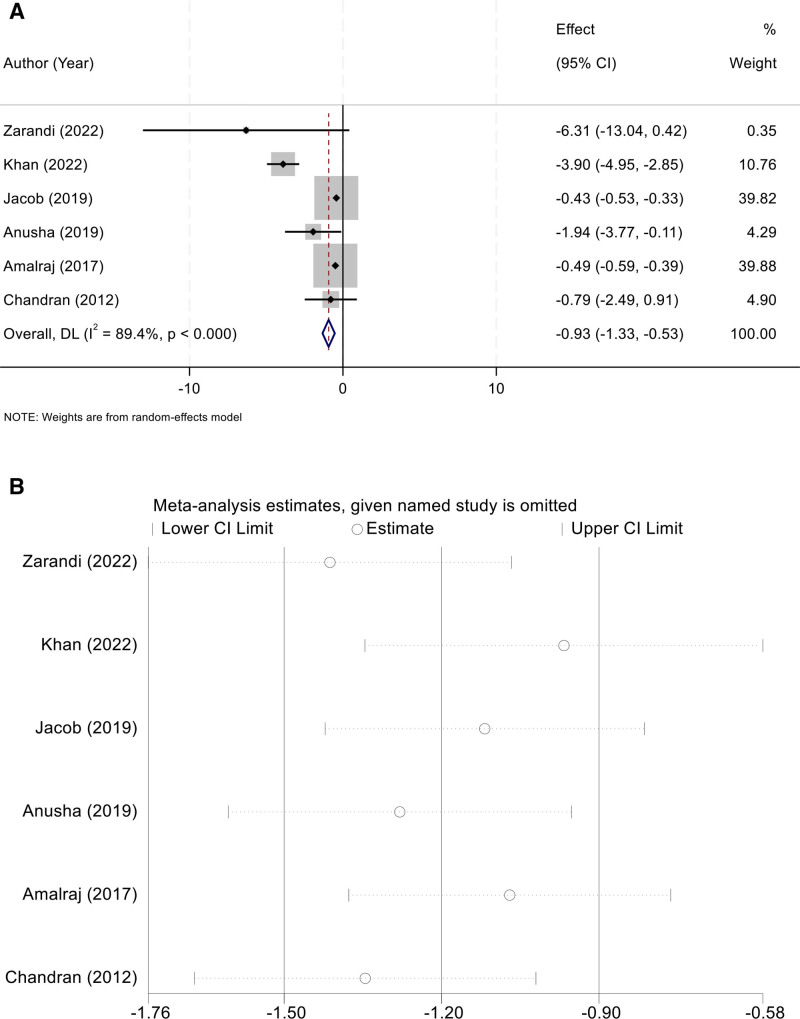
(A) Random-effects forest plot of WMD for CRP (negative favors curcumin); (B) leave-one-out sensitivity analysis showing stability of the pooled effect. CRP = C-reactive protein, WMD = weighted mean difference.

Six studies evaluated CRP levels as an outcome. Considerable heterogeneity was observed (*I*² = 89.4%, *P* < .001), and a random-effects model was applied. The results indicated that curcumin supplementation significantly reduced CRP concentrations compared with controls (WMD = –0.93, 95% CI [–1.33, –0.53]) (Fig. 6A). Sensitivity analysis confirmed the stability of the findings, with no major changes observed after sequential study removal (Fig. 6B).

### 3.8. Publication bias

The potential for publication bias was assessed using Egger linear regression test. The results suggested no significant evidence of publication bias across the included analyses (*P* > .05 for all outcomes), supporting the reliability and robustness of the meta-analytic conclusions.

### 3.9. Safety outcomes

Among the included trials, 5 studies explicitly reported adverse events, while 2 did not provide safety information. Across the available data, no serious treatment-related adverse events were observed. Mild gastrointestinal discomfort (nausea, bloating), transient headache, and mild skin rash were the most commonly reported events, occurring in both curcumin and control groups with similar frequency. No withdrawals due to adverse events were reported in any study. Overall, the available evidence suggests that curcumin was well tolerated during treatment courses of 8 to 12 weeks; however, incomplete reporting and short follow-up periods limit conclusions regarding long-term safety.

## 4. Discussion

Across randomized and controlled evidence, curcumin supplementation was associated with statistically significant improvements in both composite disease activity and inflammatory biomarkers in patients with RA. The pooled WMD for the DAS28 indicated a reduction of –1.47 (95% CI –1.68 to –1.26). Interpreted against established EULAR response thresholds, where a DAS28 decrease > 1.2 generally reflects at least a moderate, and often a good, treatment response, this magnitude suggests clinically meaningful improvement at the group level while acknowledging between-study heterogeneity in baseline disease activity and concomitant therapies.^[20]^ Consistent decreases were also observed for RF, ESR, and CRP, supporting a systemic anti-inflammatory signal. Biologically, these findings are plausible. Curcumin mainly inhibits key inflammatory pathways such as nuclear factor-kappa B and Janus kinase/signal transducer and activator of transcription, leading to reduced expression of TNF-α, interleukin-1 beta, and IL-6. These changes can suppress hepatic acute-phase protein synthesis, explaining the observed reductions in CRP and ESR, and mitigate synovial inflammation reflected in DAS28 and RF improvements. The consistency of these effects across sensitivity analyses further supports the robustness and biological plausibility of the anti-inflammatory signal.^[21,22]^

Several study-level factors may explain the heterogeneity (*I*² 89%–99% for biomarkers and 92% for DAS28). First, formulations varied widely, from conventional curcumin powders to nanomicelles and other enhanced-bioavailability systems; these platforms achieve markedly different systemic exposures and may yield different effect sizes.^[23,24]^ Second, doses and dosing schedules ranged from once-daily low milligram amounts to multiple daily administrations, with treatment durations spanning 8 to 12 weeks or 90 days, which may be insufficient for maximal disease modification in some patients. Third, background disease-modifying antirheumatic drug (DMARD) regimens and baseline disease states were not uniform, potentially interacting with curcumin’s effects. Finally, outcome ascertainment (DAS28-ESR vs DAS28-CRP) can introduce small systematic differences in absolute values and treatment-related change.^[25]^

Our results align with and extend contemporary syntheses. A 2023 systematic review reported significant reductions in ESR and CRP with curcumin versus control in RA, with heterogeneity similar in magnitude to that observed here.^[26]^ An umbrella review published in 2025 that aggregated evidence across multiple health outcomes similarly noted significant reductions in ESR and CRP in RA, citing the 2023 RA meta-analysis as the primary contributor; the effect sizes were of comparable direction and magnitude to the present findings.^[27]^ These corroborations increase confidence that the anti-inflammatory signal is not idiosyncratic to a single dataset. Recent randomized evidence further informs interpretation. A 2025 double-blind trial evaluating a nanomicelle curcumin formulation in RA reported clinical benefits (e.g., improved symptoms and immune parameters) but no statistically significant change in ESR over the trial horizon, highlighting that biomarker responses may lag or depend on formulation, exposure, and baseline inflammatory load.^[28]^ Divergent biomarker results across modern randomized controlled trials underscore that enhanced formulations do not uniformly translate to proportional changes in all endpoints within short follow-up windows, and they emphasize the importance of dose, duration, and pharmacokinetics. A central interpretive theme in the recent literature is bioavailability. Pharmacokinetic and formulation reviews from 2024 to 2025 show that adjuvants (e.g., piperine) and delivery technologies (nanoparticles, micelles, phospholipid complexes) can increase curcumin exposure by orders of magnitude, plausibly amplifying clinical signals; however, the extent of improvement and its translation to disease endpoints vary by platform and study design.^[24]^ As most trials in our synthesis did not standardize formulations or verify systemic exposure, effect modification by bioavailability remains a key uncertainty. Collectively, the 2023 to 2025 evidence base therefore supports curcumin’s potential as an adjunctive anti-inflammatory agent in RA while also calling for rigorously designed, exposure-verified trials to determine the dose–response relationship and to compare delivery systems head-to-head.

Taken together, the reductions in DAS28 and acute-phase reactants suggest that curcumin may serve as an adjunct to standard RA care for patients with persistent low-grade inflammatory activity, intolerance to dose escalation of DMARDs, or preferences for complementary strategies. In clinical practice, use should be individualized, with attention to potential drug–supplement interactions, product quality, and realistic expectations regarding magnitude and timing of benefit. Short courses (8–12 weeks) may be considered for symptom and biomarker reassessment, with continuation contingent upon demonstrable response and safety. These conclusions should complement, not replace, guideline-directed DMARD therapy. This meta-analysis synthesized randomized and controlled evidence across multiple clinically relevant outcomes, used random-effects models under high heterogeneity, and performed sensitivity analyses that supported the stability of effect estimates. Publication bias was not detected by Egger testing across outcomes, adding assurance to the observed benefits.

Several limitations warrant consideration. First, heterogeneity was substantial for most endpoints, limiting precision in pooled estimates and the ability to infer uniform effects. In particular, differences in curcumin formulations, dosages, treatment durations, and concomitant background therapies likely contributed to this heterogeneity. Because most included trials did not verify systemic exposure or report stratified results by these factors, we were unable to perform meaningful subgroup or meta-regression analyses to explore their effects. Future studies should therefore standardize formulations, define dose–exposure targets, and report treatment context in greater detail. Second, the absence of pharmacokinetic data precluded formal dose–response analyses; unmeasured variability in bioavailability remains a plausible source of between-study variance. Third, follow-up durations were short (typically ≤ 12 weeks), constraining inferences about durability of response and structural outcomes. Fourth, trial sizes were modest and often single-center; although risk-of-bias assessments were generally acceptable, incomplete outcome data or limited allocation concealment was occasional concerns. Moreover, 1 included trial recruited RA patients with comorbid chronic periodontitis, representing a specific subpopulation. Although inclusion required fulfillment of standard ACR/EULAR criteria and sensitivity analyses did not indicate undue influence on the pooled estimates, this may nonetheless limit the generalizability of the findings to the broader RA population. Fifth, variation in background DMARDs and baseline disease activity was insufficiently detailed to support subgroup analyses by concomitant therapy, severity, or serostatus. Sixth, the safety data extracted from the included studies were limited but have now been summarized separately in the Results section to provide a balanced assessment of efficacy and tolerability. No serious treatment-related adverse events were reported, though the short duration of follow-up restricts conclusions about long-term safety. Finally, differences in DAS28 computation methods (ESR vs CRP) may yield measurement heterogeneity that future trials should standardize a priori.

Future trials should prioritize exposure-verified dosing using standardized, high-quality formulations with documented pharmacokinetics; adequately powered, multicenter designs with longer follow-up to evaluate durability, remission, and steroid-sparing effects; prespecified subgroups by baseline disease activity, serology, and concomitant DMARD/biologic therapy; and harmonized outcome sets including DAS28-ESR and DAS28-CRP (or alternative indices), patient-reported outcomes, and safety endpoints. Pragmatic trials embedded in routine care and comparative studies of bioavailability-enhanced delivery systems would be particularly informative. In parallel, mechanistic studies should quantify curcumin’s in vivo effects on cytokine networks and B-cell autoantibody pathways in RA.

## 5. Conclusions

Curcumin supplementation was associated with significant reductions in DAS28, RF, ESR, and CRP versus control, and these findings were robust to sensitivity analyses. Despite considerable interstudy heterogeneity, likely related to formulation, dose, and short follow-up, the overall signal supports curcumin as a potential adjunct to guideline directed RA therapy. Larger, exposure-verified, longer term randomized trials are needed to define optimal regimens, durability, and safety.

## Author contributions

**Conceptualization:** Fuhan Zhang, Ben Niu.

**Data curation:** Fuhan Zhang, Ben Niu.

**Formal analysis:** Fuhan Zhang.

**Funding acquisition:** Ben Niu.

**Investigation:** Fuhan Zhang, Ben Niu.

**Methodology:** Fuhan Zhang, Ben Niu.

**Supervision:** Fuhan Zhang, Ben Niu.

**Validation:** Fuhan Zhang, Ben Niu.

**Visualization:** Fuhan Zhang, Ben Niu.

**Writing – original draft:** Fuhan Zhang, Ben Niu.

**Writing – review & editing:** Fuhan Zhang, Ben Niu.

## Supplementary Material


