# Supplementary material for: Effect of curcumin on inflammatory markers and disease activity in patients with rheumatoid arthritis: A meta-analysis
Source: Medicine (Baltimore). 2025 Nov 28;104(48):e46177. doi: 10.1097/MD.0000000000046177 (PMC12662462; doi:10.1097/MD.0000000000046177)
Supplement: Supplementary file 1 [file medi-104-e46177-s001.docx]

Supplementary Table 1 Detailed Search Strategies for Each Database.

| Database | Search Strategy |
| --- | --- |
| PubMed | (("Curcumin"[Mesh] OR curcumin[tiab] OR turmeric[tiab] OR "Curcuma longa"[tiab]) AND ("Arthritis, Rheumatoid"[Mesh] OR "rheumatoid arthritis"[tiab] OR RA[tiab]) AND ("Randomized Controlled Trial"[Publication Type] OR "Controlled Clinical Trial"[Publication Type] OR randomized[tiab] OR randomly[tiab] OR placebo[tiab] OR trial[tiab])) |
| Embase | ('curcumin'/exp OR curcumin:ti,ab OR turmeric:ti,ab OR 'curcuma longa':ti,ab) AND ('rheumatoid arthritis'/exp OR 'rheumatoid arthritis':ti,ab OR RA:ti,ab) AND ('randomized controlled trial'/exp OR 'controlled clinical trial'/exp OR random*:ti,ab OR placebo:ti,ab OR trial:ti,ab) |
| Web of Science | TS=(curcumin OR turmeric OR "Curcuma longa") AND TS=("rheumatoid arthritis" OR RA) AND TS=(randomized OR randomly OR "controlled trial" OR placebo OR trial) |
| Cochrane Library | (curcumin OR turmeric OR "Curcuma longa") in Title Abstract Keyword AND ("rheumatoid arthritis" OR RA) in Title Abstract Keyword AND (randomized OR randomly OR "controlled trial" OR placebo OR trial) in Title Abstract Keyword |
